# Processing of Snake Venom Metalloproteinases: Generation of Toxin Diversity and Enzyme Inactivation

**DOI:** 10.3390/toxins8060183

**Published:** 2016-06-09

**Authors:** Ana M. Moura-da-Silva, Michelle T. Almeida, José A. Portes-Junior, Carolina A. Nicolau, Francisco Gomes-Neto, Richard H. Valente

**Affiliations:** 1 Laboratório de Imunopatologia, Instituto Butantan, São Paulo CEP 05503-900, Brazil; michelle.almeida@butantan.gov.br (M.T.A.); portes.junior@butantan.gov.br (J.A.P.-J.); 2 Laboratório de Toxinologia, Instituto Oswaldo Cruz, Rio de Janeiro CEP 21040-360, Brazil; carolnicolau.bio@gmail.com (C.A.N.); gomes.netof@gmail.com (F.G.-N.); richardhemmi@gmail.com (R.H.V.)

**Keywords:** snake venom, metalloproteinase, post-translational processing, enzyme inhibitor, hemorrhage

## Abstract

Snake venom metalloproteinases (SVMPs) are abundant in the venoms of vipers and rattlesnakes, playing important roles for the snake adaptation to different environments, and are related to most of the pathological effects of these venoms in human victims. The effectiveness of SVMPs is greatly due to their functional diversity, targeting important physiological proteins or receptors in different tissues and in the coagulation system. Functional diversity is often related to the genetic diversification of the snake venom. In this review, we discuss some published evidence that posit that processing and post-translational modifications are great contributors for the generation of functional diversity and for maintaining latency or inactivation of enzymes belonging to this relevant family of venom toxins.

## 1. Introduction

Generation of diversity is a very important feature in the evolution of different species of animals, especially in systems in which fast adaptation to the environment is required. The most relevant system in which the generation of diversity plays a key role is the immune system. A large repertoire of antibody molecules, T cell receptors, and MHC antigens are mostly generated by intrinsic mechanisms of genetic recombination, together with post-transcriptional and post-translational processing [1,2,3,4,5,6,7], generating molecules responsible for host protection against aggressors and for self-maintenance. Although such a large repertoire is not necessary for many other systems, generation of diversity is also used as mechanism for fitness enhancement in most venomous animals, from cone snails [8] to advanced snakes [9], generating a toxin array that interacts with functionally-relevant receptors of different species [10], enabling capture of a greater diversity of prey, or evasion from different predators. In advanced snakes, generation of diversity of venom components was a great adaptive advantage that allowed the radiation of several taxa after the appearance of the venom glands, recruitment and neofunctionalization of toxin genes, and development of the venom injection system [9,10]. A few gene families have been recruited for snake venom production [11]. However, these genes are under accelerated evolution and undergo a number of duplications followed by distinct genetic modification mechanisms as accumulation of substitutive mutations, domain loss, recombination, and neofunctionalization that result in the large diversity within venom toxin gene families [12,13,14,15,16,17].

Snake venom metalloproteinases (SVMPs) are particularly important for the adaptation of snakes to different environments. In the venoms of most species of viper snakes, SVMPs are the most abundant component [18,19] and, as discussed above, the evolutionary mechanisms of this gene family allowed the structural and functional diversity of SVMPs in viper venoms. SVMPs are able to interact with different targets that control hemostasis or relevant tissues related to essential physiological functions in prey and predators [20,21]. The most evident effect of SVMPs is hemorrhage, as a result of a combined disruption of capillary vessels integrity and impairment of the blood coagulation system, resulting in consumption of coagulation plasma factors [20]. The mechanisms of action of distinct SVMPs involve different targets as, for example, activation of coagulation Factor X [22], activation of Factor II [23], fibrino(gen)olytic activity [24], binding and damage of capillary vessels [25,26,27], among others. SVMPs interacting with distinct hemostatic targets may be found in the same pool of venom from a single species [21] and, together, these different enzymes interfere with the whole hemostatic system, subduing prey usually by shock [28].

The structural diversity of SVMPs is well known [29,30,31] and three classes (P-I, P-II, and P-III), further subdivided into at least 11 subclasses, have been described based on their domain structure [31]. This classification is based on the presence of different domains in the zymogens predicted by the mRNA sequences and the mature form of the enzymes. They are synthesized as pro-enzymes with pre- and prodomains responsible for directing the nascent proteins to the endoplasmic reticulum and for maintaining the latency of the enzyme before secretion, respectively [32,33]. The mechanism involved in the activation of SVMPs (step of biosynthesis involving the removal of the prodomain) is still understudied. Furthermore, an eventual role played by the free prodomain (or its fragments) in enzyme activity after secretion is elusive. In addition to pre- and prodomains, a catalytic domain is present in P-I, P-II, and P-III classes at the C-terminus of the prodomain and is the only domain present in mature class P-I SVMPs. P-II and P-III SVMP classes differ from the former by the presence of non-catalytic domains included at the C-terminus of the catalytic domain: the disintegrin domain in P-II class and disintegrin-like plus cysteine-rich domains in the P-III class [31].

Genes coding for P-III class SVMPs appear to have been the first recruited to the snake venom while P-II and P-I SVMP genes appeared in viperids later, mostly by domain loss [16]. However, genetic mechanisms are not the only ones responsible for generating diversity in SVMPs. Due to the recent increase of data generated by venom proteomes and transcriptomes, it has also become evident that post-transcriptional [34] and post-translational [35] modifications represent additional sources of diversity generation in venom composition, increasing the possibilities of mechanisms of predation and resulting in an adaptive advantage for snakes. In this review, we will focus on the role of processing of nascent SVMPs in the generation of diversity and in the inactivation of these enzymes during and after their biosynthesis.

## 2. Biosynthesis and Post-Translational Processing of SVMPs

**Figure 1:**
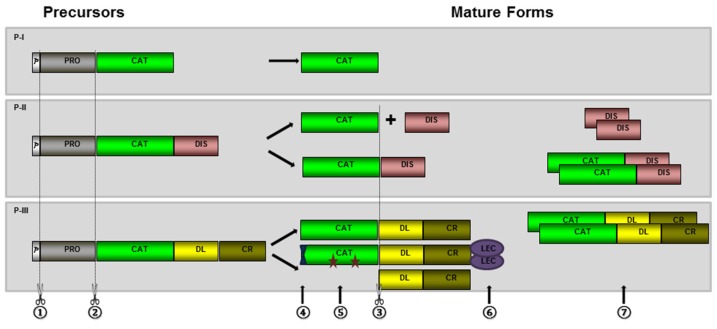
Schematic representation of the most typical post-translational modification steps occurring during the maturation of nascent SVMPs: SVMP precursors are composed of signal-peptide/pre- (p), pro- (PRO), catalytic or metalloproteinase (CAT), disintegrin (DIS), disintegrin-like (DL), cysteine-rich (CR), and lectin-like (LEC) domains. Processing of nascent SVMP involves removal of signal-peptide/pre-domain (①) hydrolysis of the prodomain (②) and disintegrin or disintegrin-like/cysteine-rich domains (③), cyclization of amino-terminal glutamyl residues to pyro-glutamate (④), glycosylation (represented by stars—⑤), addition of new domains (⑥) or dimerization of protein chains (⑦).

**Figure 2:**
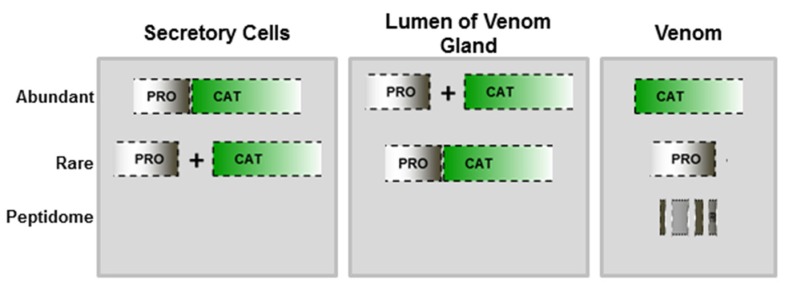
Schematic representation of prodomain processing: Antibodies against jararhagin prodomain detected predominantly bands of zymogen molecular mass in secretory cells and processed form in the lumen of the venom gland. Prodomain was poorly detected in the venom, suggesting that SVMPs are mostly in the active form.

**Figure 3:**
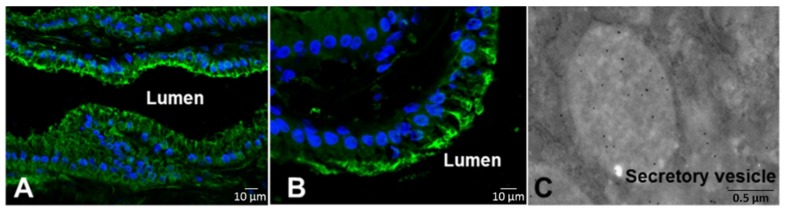
Cellular localization of prodomains. Venom glands collected before (**A**) or seven days after (**B**, **C**) venom extraction were sectioned and subjected to immunofluorescence (**A**, **B**) stained with DAPI (blue) and mouse anti-PD-Jar serum (green), which concentrated in the apical region of secretory cells, or electron microscopy (**C**) after staining with anti-PD-Jar serum, which highlighted spots in the secretory vesicles [46].

SVMP transcripts predict proteins with multi-domain structure that undergo different post-translational processing generating distinct mature proteins (Figure 1). As other secreted proteins, SVMPs include a signal-peptide/predomain (p) responsible for driving the nascent SVMP to the endoplasmic reticulum where most of the protein modifications take place. In the endoplasmic reticulum, the pre-domain is removed by signal peptidases (Figure 1-①) resulting in zymogens that are subjected to further modifications [36]. Activation of the enzymes occurs by hydrolysis of the prodomain (Figure 1-②) [37] and, after this step, disintegrin or disintegrin-like/cysteine-rich domains can also be released by proteolysis (Figure 1-③) [38]. Mature forms may present other modifications such as cyclization of amino-terminal glutamyl residues to pyro-glutamate (Figure 1-④), glycosylation (Figure 1-⑤), addition of new domains (Figure 1-⑥), or dimerization of protein chains (Figure 1-⑦) [36]. These steps occur to different extents depending on the primary structure of the precursors predicted by the paralogue genes that originate the transcripts. As a result, different biological activities are associated to each particular isoform. Therefore, post-translational modifications are essential for activity and stability of the proteins, and also for diversifying their specific targets. Some of the issues related to each of these processing steps will be discussed below.

### 2.1. Hydrolysis of the Prodomain

Activation of SVMPs is regulated by hydrolysis of their prodomains, as happens with matrix metalloproteinases (MMPs) and disintegrin and metalloproteinase (ADAM) proteins. Prodomains of SVMPs include a conserved motif (PKM**C**GVT), also found in ADAM and MMP precursors [33]. In this motif, a free cysteine residue is a key factor for maintaining enzyme latency via a cysteine-switch mechanism. This process controls the activation state of enzymes by blocking the catalytic site (inactivated state) before the proteolytic processing of the prodomain (active state) [33,39].

In MMPs, activation generally occurs at the extracellular space catalyzed by members of the plasminogen/plasmin cascade, by other MMPs or by chemical modification of the conserved cysteine residue in the cysteine switch motif [40,41,42]. A different mechanism of activation is verified in ADAMs, as the prodomain is generally removed intracellularly by pro-protein convertases [43], or by autocatalytic mechanisms [31,44,45]. In SVMPs, only a few studies attempted to explain enzyme activation and/or hydrolysis of their prodomains. However, studying the activation of recombinant pro-atrolysin-E, Shimokawa and collaborators [38] suggested that chemical modifications are not efficient for activation, which probably occurs by proteolysis by metalloproteinase present on the crude venom. In a recent study from our group [46], we used antibodies specific to jararhagin prodomain to search for the presence of prodomains in different compartments of snake venom glands, either as zymogens or in the processed form. Using gland extracts obtained at different times of the venom production cycle, we immunodetected electrophoretic bands matching to the SVMP zymogen molecular mass (in high abundance), and only faint bands with molecular masses corresponding to different forms of cleaved prodomain. However, the presence of zymogens in the venom was rare, detected only as faint bands in samples collected from the lumen of venom gland at the peak of venom production. In milked venom, only weak bands corresponding to free prodomain were detected in samples collected at the peak of venom production, suggesting that most of the prodomain molecules promptly undergo further hydrolysis, generating diverse peptides that are not immunoreactive with anti-PD-Jar (Figure 2). In agreement to this suggestion, SVMP prodomain peptides are very rare in proteomes of viper venoms, with a few exceptions [47], but they were recently found in the proteopeptidome of *B. jararaca* venom [48], and in proteomes of *B. jararaca* gland extracts [49], which is consistent with our hypothesis.

Using immunohistochemistry and immunogold electromicroscopy, prodomain detection was concentrated in secretory vesicles of secretory cells (Figure 3). According to these images, we suggested that SVMPs are stored at secretory cell vesicles mostly as zymogens; the processing of prodomains starts within the secretory vesicles but reaches its maximal level during secretion or as soon as it reaches the lumen of the venom gland.

According to these data, processing and activation of SVMPs undergo distinct routes than MMPs or ADAMs. MMPs are critical enzymes for remodeling the extracellular matrix in a series of physiological and pathological processes as angiogenesis, wound healing, inflammation, cancer, and infections [40,50]. The regulatory role of MMPs in such processes requires a well-controlled mechanism of activation for which the secretion of latent enzymes is of great advantage. On the other hand, most ADAMs are transmembrane proteins that regulate mostly cell migration, adhesion, signaling and, eventually, proteolysis. In this case, processing of ADAMs through the secretion pathway by furins and other processing enzymes is the most common processing route [43,51]. SVMPs apparently undergo different processing routes since the release of the prodomain is very likely to occur during the secretion of vesicle contents. The enzymes responsible for the processing have not yet been identified, but it can be speculated that venom serine proteinases or even metalloproteinases could be involved. Moreover, a series of convertases have been detected in proteomic and transcriptomic studies [49]. One issue that remains unsolved and will be discussed below is whether SVMPs are maintained in the lumen of the venom gland in the active form or are kept inactivated by peptides liberated by prodomain hydrolysis or by other inhibitory factors present in the venom as the acidic pH environment, high citrate concentrations and tripeptides containing pyroglutamate.

### 2.2. Generation of Disintegrin and Disintegrin-Like Domains

Disintegrins are generated by proteolysis of SVMPs originated from class P-II transcripts. These small molecules are abundant in venoms of viper snakes that usually contain the RGD or a related (XGD) motif in a surface exposed loop that binds to RGD-dependent integrins, such as α_IIb_β_3_, α_5_β_1_, and α_v_β_3_ or, in a few cases, they may display a MLD motif, targeting α_4_β_1_, α_4_β_7_, and α_9_β_1_, or a K/RTS motif that is very selective for binding to α_1_β_1_ integrin [52]. These are important receptors of different cell types, particularly platelets, inflammatory, and vascular endothelial cells, in which they are responsible for inhibition of platelet aggregation or endothelial cell adhesion, migration, and angiogenesis [53,54]. For these reasons, the inclusion of disintegrins in the venom of viper snakes conferred a great adaptive advantage for using hemostatic targets to surrender prey.

Genes coding for class P-II SVMPs have evolved from P-III ancestor genes by a single loss of the cysteine-rich domain followed by convergent losses of the disintegrin domain at different phylogenetic branches that were further responsible for the generation of distinct P-I SVMP structures [16]. After the cysteine-rich domain loss, evolution of P-II genes was continued by gene duplication and neofunctionalization of the disintegrin domain in some of the duplicated copies [16,55]. Other genetic mechanisms of recombination as exon shuffling or pre- or post-transcriptional recombination could also play a role in the diversification of class P-II SVMP structures. The first draft of the genomic organization of a PIII-SVMP gene revealed a series of nuclear retroelements and transposons within introns that could provide genomic explanations for the emergence of distinct class P-II messengers [56]. Evidence for post-transcriptional modification arose when *B. neuwiedi* SVMP cDNA sequences were analyzed: three distinct types of P-II sequences were noted including a typical transcript of class P-II SVMP and other transcripts that presented clear indications of recombination between P-II disintegrin domain coding regions with either P-I or P-III catalytic domain coding regions [34]. The data suggest that recombination between genes encoding SVMPs might have occurred after the emergence of the primary gene copies coding for each scaffold. Moreover, it has also been reported by different authors the occurrence of SVMP structures that might have been assembled by the P-III catalytic domain with the P-II disintegrin domain [57] or even by PII catalytic domains with the P-III disintegrin-like domains lacking the cysteine-rich domain [58]. Unfortunately, these mechanisms of recombination are still speculative since, up to now, genomic sequences coding for SVMPs were not completely disclosed and the exon/intron distribution at catalytic domain is still unknown.

In viper venoms, the products of P-II genes are diverse and the precursors undergo proteolytic steps depending on the structure predicted by the paralogue gene coding for each different toxin. Most P-II precursors are hydrolyzed at the spacer region, located between catalytic and disintegrin domains [31] generating free disintegrins and catalytic domains that are frequently found in venoms, and also recognized as classical disintegrins and P-I class SVMPs, respectively. However, some P-II precursors are not hydrolyzed and are expressed in the venom as single chained molecules, containing catalytic and disintegrin domains. The enzymes involved in the cleavage of P-II precursors to generate free disintegrins are still unrecognized and the mechanisms by which different P-II precursors are processed (or not) are still speculative. A mainstream hypothesis, postulated by Serrano and Fox [31], suggests that the presence of cysteinyl residues, particularly at the spacer region and at the *N*-terminus of the disintegrin domain, would confer more resistance to hydrolysis, acting in favor of maintenance of P-II SVMPs in the catalytic form. A few of these enzymes have been characterized [53,59,60,61] and recent reports indicate potent hemorrhagic activity in catalytic P-II SVMPs that may be achieved by their capability to cleave ECM proteins combined to their potential to inhibit platelet aggregation and/or to bind to basal lamina [62].

Some P-III class SVMPs are also cleaved generating fragments which correspond to the disintegrin-like/cysteine-rich domains [31]. However, cleavage mechanisms and the fate of the domains after cleavage are apparently different than the ones observed in P-II SVMPs. Most P-III SVMPs are found in venoms in their multi-domain form, containing catalytic, disintegrin-like and cysteine-rich domains, although examples of autolysis at the spacer region of isolated P-III SVMPs have been reported for jararhagin, from *Bothrops jararaca* [63], HR1A and HR1B from *Trimeresurus flavoviridis* [64], HT-1 from *Crotalus ruber ruber* [64], brevilysin H6, from *Gloydius halys brevicaudus* [65], alternagin, from *Bothrops alternatus* [66], batroxhagin, from *Bothrops atrox* [67,68], and catrocollastatin, from *Crotalus atrox* [69]. Autolysis of these proteins usually results in combined disintegrin-like/cysteine-rich domain fragments, known as “C” proteins, which are characterized as inhibitors of collagen-induced platelet-aggregation [63], but may also display pro-inflammatory activity [70] or stimulate endothelial cells to release pro-angiogenic mediators [71]. On the other hand, the free catalytic domain that results from this autolytic process has never been found in venoms, suggesting that a fast hydrolysis of the catalytic domain to small peptides occurs after autolysis. In *B. jararaca* venom, the presence of both intact jararhagin and processed jararhagin-C is currently detected [63,72]. Moreover, a third form of processed protein was detected that comprised a processed form of jararhagin-C linked to the catalytic domain by disulfide bonds [73]. This evidence suggests that at least three different proteoforms of jararhagin may exist and they probably display distinct pairing of cysteinyl residues that will drive to three different autolytic pathways. The three possibilities of autolysis appear to occur in venoms of other snakes. We recently reported the same three forms of processed batroxrhagin, a P-III SVMP isolated from the venom of *B. atrox* [67]. The presence of these different forms of P-III SVMPs in venoms contributes to greater structural and functional complexity of the venom, and may be a common feature among other class P-III SVMPs.

### 2.3. Dimerization and Inclusion of Other Domains

The position and pairing of cysteinyl residues certainly play a role in the liberation of disintegrins, but they are also very important in generating multimeric structures of some nascent SVMPs, increasing their structural and functional diversity. One example is the linkage of lectin-like domains to the cysteine-rich domain of class P-III SVMPs generating very active pro-coagulant toxins as RVV-X, from *Vipera russelli* [32,33,34], classified as a PIIId, or previously as a P-IV SVMP [30]. However, homodimers or heterodimers of homologous domains are most commonly found in the venoms and dimerization apparently contributes to the enhancement of the toxin activity. Class P-III SVMPs VAP1 and VAP2 from *Crotalus atrox* have been crystallized in their dimeric form [74,75] exerting potent pro-apoptotic activity in endothelial cell cultures [76]. Bilitoxin and BlatH1 are also examples of non-processed P-II SVMP homodimers [77,78]. Interestingly, in both cases the RGD sequence displayed in the disintegrin domain is replaced by MGD and TDN, respectively, resulting in toxins that are unable to block the platelet fibrinogen receptor; however, both dimeric P-II SVMPs are potent hemorrhagins with activity levels comparable to those of class P-III SVMPs [62,77].

The most common dimers of SVMPs present in venoms are undoubtedly homo- and heterodimeric disintegrins. For example, contortrostatin, a homodimeric disintegrin that displays RGD motifs in both chains, has been produced in a recombinant form [79] and presented substantial anti-angiogenic and anti-cancer effects [79] with some efficacy as an adjuvant in chemotherapy for melanoma [80] and for viral infections [81]. Heterodimers composed of distinct disintegrin domains have also been isolated. Usually, one chain contains the conserved RGD motif and the other chain displays alternative motifs that modulate specificity and selectivity. The first heterodimeric disintegrin group identified included EMF-10 and CC-8 disintegrins, with a RGD motif in one chain and a WGD in the other [82]. As a result, the heterodimer is the strongest blocker of the fibrinogen receptor and effective to modulate megakaryocyte activity [83]. Other interesting group of heterodimeric disintegrins includes EC3, VLO5, and EO5 with V/RGD motif in one chain and the MLD motif in the other [84]. These toxins target mainly α_4_β_1_, α_4_β_7_, and α_9_β_1_ integrins, mostly related to inflammatory cell receptors [85]. Heterodimeric disintegrins are common in venoms of *Viperinae* subfamily of vipers, and the substitution of the RGD motif at least in one chain may decrease the effect of these toxins on platelets related to impairment of hemostasis. Although this is an apparent disadvantage for the snakes, the non-RGD disintegrins are undoubtedly good leading molecules for drug development or for producing biotechnological tools to address the mechanisms of action of integrin receptors.

### 2.4. Other Post-Translational Modifications

Most of snake venom proteins undergo glycosylation during their biosynthesis pathway. In eukaryotic cells, glycosylation influences important biochemical properties of the proteins, such as folding, stability, solubility, and ligand binding. In spite of the importance of glycosylation for eukaryotic-secreted proteins, very little is known about the carbohydrate structures present in venom glycoproteins.

In SVMPs, primary structures predict several putative *N*-glycosylation sites [31], and important functions have already been correlated to glycan moieties. However, glycosylation sites identified in the available crystal structures of SVMPs indicate a significant variability, and suggest that the presence of glycan moieties is not predictable based on primary structure information only [86]. Studying the cobra venom glycome, Huang and co-authors [86] identified four major *N*-glycan moieties on the biantennary glycan core, and a high variability of *N*-glycan composition in SVMPs from individual snake specimens. In the same study, the authors reported that these glycoproteins elicit much higher antibody response in antiserum when compared to other high-abundance cobra venom toxins, such as small molecular mass CTXs. The higher immunogenicity of SVMPs compared to other venom components has been also shown by our group [18], and it can be at least partially attributed to the glycan moieties present on these molecules. Moreover, *Bothrops jararaca* SVMPs bothropasin, BJ-PI, and HF3 were subjected to *N*-deglycosylation that induced loss of structural stability of bothropasin and BJ-PI. Although HF3 remained apparently intact, its hemorrhagic and fibrinogenolytic activities were partially impaired, suggesting the importance of glycans for stability, and also for the interaction with substrates [87].

The role of glycosylation in the generation of toxin diversity has been recently addressed in a few studies approaching ontogenetic or gender-related venom variability. The *N*-glycan composition of newborn and adult venoms did not vary significantly [88], but gender-based variations contributed to different glycosylation levels in toxins [35]. The studies demonstrated a complexity of carbohydrate moieties found in glycoproteins, indicating another level of complexity in snake venoms that could be related to the diversification of biological activities.

Another form of post-translational modification observed in many SVMPs is the cyclization of amino-terminal glutaminyl residues to pyro-glutamate. The cyclization of glutaminyl residues by the acyltransferase glutaminyl cyclase is a common occurrence in many organisms. For many bioactive peptides, cyclization of amino-terminal glutaminyl residues renders the peptide resistant to proteolytic processing by exopeptidases, thus protecting their biological activities [89]. Glutaminyl cyclase has been identified in the venom of viperid snakes [90] particularly in venoms collected at the seventh and tenth days after venom extraction [46]. Most class P-III SVMPs possess, in their mature form, a pyroglutamic acid as the *N*-terminus [31], and this modification provides protection to these enzymes from further digestion by aminopeptidases, or even further processing steps resulting in the release of disintegrin domains.

## 3. The Role of Prodomains for Enzyme Inactivation

One still-unsolved issue is the maintenance of mature enzymes in the lumen of the venom gland. Since SVMPs degrade extracellular matrix components [91,92] and can lead to loss of viability of different cell types [20] (including epithelial cells [93]), they can be considered a potential risk for the maintenance of venom gland integrity. It is currently accepted that the acidic pH environment in the lumen of the venom gland could limit proteolytic activity of SVMPs [94]. Additionally, high citrate concentrations and tripeptides containing pyroglutamate found in venoms could inhibit SVMPs that would be activated after venom injection due to dilution factors [95,96]. Previously, secretion of SVMPs into the lumen of the gland as zymogens was also considered as a mechanism for maintaining the latency of these enzymes. However, we have recently shown that the activation of SVMPs mainly occurs during the secretion to the lumen of the venom gland, and cleaved prodomains undergo further hydrolysis to small peptides [46].

The potential of peptides containing the cysteine-switch motif for the inactivation of SVMPs has already been shown [37]. This work led us to test if processed prodomain or prodomain degradation peptides could play a role in the inhibition of activated SVMPs, within the gland environment. To test this hypothesis, we produced jararhagin recombinant prodomain (PD-Jar) as described [46], and synthesized a 14-mer *C*-terminally amidated peptide (SynPep), based on a naturally-occurring prodomain peptide fragment that was abundantly detected in *Bothrops jararaca* peptidome [48]. Interestingly, our preliminary data show that both recombinant PD-Jar and SynPep inhibited jararhagin catalytic activity, and also toxic activities such as induction of fibrinolysis and hemorrhage (Table 1).

Inhibition of metalloproteinase activity by isolated prodomains has been recently addressed in the search for specific therapeutic tools for pathologies involving these enzymes. The cleaved prodomain of certain ADAMs can act as a selective inhibitor of the catalytic activity of the enzyme. Moss and coworkers [99] and Gonzales *et al.* [100] produced the recombinant prodomains of ADAM-9 and TACE, respectively, for the purpose of understanding their mechanism of inhibition and selectivity against these proteinases. In these studies, ADAM-9 prodomain was highly specific and the inhibition of ADAM-9, by its recombinant prodomain, regulated ADAM-10 activity controlling the release of soluble α-secretase enzyme, which is an important task in the therapy Alzheimer's disease [99]. Additionally, TACE prodomain was also specific for this enzyme and could be used as a potential inhibitor of TNF-α release in inflammatory diseases [100]. Considering the high selectivity of prodomains as metalloproteinase inhibitors, we are currently testing more accurately the selectivity and kinetics parameters of SVMPs´ inhibition by PD-Jar and SynPep, and their effects on neutralization of local effects induced by viper venoms. These experiments may increase our understanding about the maintenance of inactivated SVMPs inside the venom glands, and could also lead to therapeutic alternatives to minimize local damage induced by snake venoms.

The animal protocols used in this work were evaluated and approved by the Animal Use and Ethic Committee (CEUAIB) of the Institute Butantan (Protocol 1271/14). They are in accordance with COBEA guidelines and the National law for Laboratory Animal Experimentation (Law no. 11.794, 8 October 2008).

## 4. Conclusions

The contribution of post-translational processing to the generation of venom diversity has been a recent issue offering important insights for understanding the complexity of animal venom arsenals. In this review, we present some current data that support the participation of post-translational processing for generation of diversity of snake venom metalloproteinases. Furthermore, there are other snake venom toxin families (serine proteinases, phospholipases, and *C*-type lectin-like proteins) represented by several proteoforms, and we predict that similar features discussed here for SVMPs could also be applicable to account, at least in part, for their diversity, and that the same would hold true for venom components from different animal species. Indeed, in a very elegant study, Dutertre and collaborators [101] explained the expanded peptide diversity in the cone snail *Conus marmoreus* revealing how a limited set of approximately 100 transcripts could generate thousands of conopeptides in the venom of a single species. More recently, Zhang and collaborators [102] working with peptide toxins from the tarantula *Haplopelma hainanum* went further into this aspect by showing the role of post-translational modifications in the generation of venom diversity and also in diversifying the functional venom arsenal. In this review, we addressed this issue for snake venom metalloproteinases. Although genetic mechanisms are essential for generating a great number of SVMP paralogue genes, post-translational processing appears as an important contributor for diversifying the SVMP arsenal able to interact with a greater number of physiological targets present in different prey. The articles revised in this paper may represent only the tip of the iceberg explaining SVMPs diversity. With analytical methods improvements, such as top-down proteomic approaches, characterization of proteoforms of complex molecules may present a larger number of possibilities for post-translational modification in SVMPs, supporting the role of processing for the stability, maintenance and functional diversification of this important toxin family present in snake venoms.

## Figures and Tables

**Table 1:** Inhibition of jararhagin activities by its recombinant prodomain (PD-Jar) or a prodomain degradation peptide (SynPep).

Activity	PD-Jar ^1^		SynPep ^1^	
	Molar Ratio	% Inhibition	Molar Ratio	% Inhibition
Catalytic ^2^	1:10	98	1:5000	90
Fibrinolytic ^3^	1:14	100	1:200	100
Hemorrhagic ^4^	1:9	100	1:500	100

^1^ Values correspond to enzyme to PD-Jar/SynPep molar ratios that resulted in inhibition of jararhagin activity; ^2^ Inhibition of enzymatic activity was tested by incubation with Abz-A-G-L-A-EDDnp as fluorescence quenched metalloproteinase substrate and compared according to the relative fluorescence units (RFU/min/µg) of each reaction [97]; ^3^ Inhibition of jararhagin fibrinolytic activity was calculated by measuring the hydrolysis halo in fibrin-containing agarose plates [98]; ^4^ Hemorrhage levels were calculated by measuring the hemorrhagic area 30 min after intradermal injection in the dorsal region of four mice [98].

## Abbreviations

The following abbreviations are used in this manuscript:

SVMP	Snake Venom Metalloproteinase
ADAM	A Disintegrin and Metalloproteinase
MMP	Matrix Metalloproteinase
PD-Jar	Recombinant prodomain of Jararhagin
MHC	Major Histocompatibility Complex
ECM	Extracellular Matrix
TACE	Tumor Necrosis Factor-alpha Converting Enzyme
DAPI	4’,6-diamidino-2-phenylindole
SynPep	Synthetic Peptide of a prodomain hydrolysis product found in *B. jararaca* venom peptidome
